# Whole Body MRI in the Detection of Lymph Node Metastases in Patients with Testicular Germ Cell Cancer

**DOI:** 10.3390/life12020212

**Published:** 2022-01-29

**Authors:** Vassiliki Pasoglou, Sandy Van Nieuwenhove, Julien Van Damme, Nicolas Michoux, Aline Van Maanen, Laurence Annet, Jean-Pascal Machiels, Bertrand Tombal, Frederic E. Lecouvet

**Affiliations:** 1 Department of Radiology, Institut Roi Albert II Cancer Center, Cliniques Universitaires Saint-Luc & Institut de Recherche Expérimentale et Clinique (IREC), Pôle IMAG, Université Catholique de Louvain (UCLouvain), 1200 Brussels, Belgium; sandy.vannieuwenhove@saintluc.uclouvain.be (S.V.N.); nicolas.michoux@saintluc.uclouvain.be (N.M.); laurence.annet@saintluc.uclouvain.be (L.A.); frederic.lecouvet@saintluc.uclouvain.be (F.E.L.); 2 Division of Urology, Cliniques Universitaires Saint-Luc, Université Catholique de Louvain (UCLouvain), 1200 Brussels, Belgium; julien.vandamme@saintluc.uclouvain.be (J.V.D.); bertrand.tombal@saintluc.uclouvain.be (B.T.); 3 Statistical Support Unit, Institut Roi Albert II Cancer Center, Cliniques Universitaires Saint-Luc, 1200 Brussels, Belgium; aline.vanmaanen@saintluc.uclouvain.be; 4 Department of Medical Oncology, Institut Roi Albert II Cancer Center, Cliniques Universitaires Saint-Luc & Institut de Recherche Expérimentale et Clinique (IREC), Pôle MIRO, Université Catholique de Louvain (UCLouvain), 1200 Brussels, Belgium; jean-pascal.machiels@uclouvain.be

**Keywords:** testicular germ cell cancer (TGCC), lymph node metastasis, staging, whole body MRI, MRI, computed tomography

## Abstract

Whole-Body Magnetic Resonance Imaging (WB-MRI) is increasingly used for metastatic screening in oncology. This prospective single center study assesses the diagnostic value of WB-MRI including diffusion weighted imaging (DWI) and identifies the sufficient protocol for metastatic lymph node detection in patients with testicular germ cell cancer (TGCC). Forty-three patients underwent contrast enhanced thoraco-abdominopelvic CT (TAP-CT) and WB-MRI with DWI for metastatic lymph node screening. Two independent readers reviewed CTs and WB-MRIs. The diagnostic performance of different imaging protocols (CT, complete WB-MRI, T1W + DWI, T2W + DWI), the agreement between these protocols and the reference standard, the reproducibility of findings and the image quality (Signal and contrast to Noise Ratios, Likert scale) were studied. Reproducibility was very good regardless of both lesion locations (retroperitoneal vs distant lymph nodes, other lesions) and the reader. Diagnostic accuracy of MRI was ≥95% (regardless of the locations and imaging protocol); accuracy of CT was ≥93%. There was a strict overlap of 95% CIs associated with this accuracy between complete WB-MRI, T1W + DWI and T2W + DWI, regardless of the reader. Higher Likert score and SNR were observed for DWI, followed by T2W and T1W sequences. In conclusion, a fast WB-MRI protocol including T2W and DWI is a sufficient, accurate, non-irradiating alternative to TAP-CT for metastatic lymph node screening in TGCC.

## 1. Introduction

Testicular germ cell cancer (TGCC) account for 1–3% of all cancers in men, being most common in the age group 15 to 35 years and considered curable in more than 90% of cases [1,2]. TGCCs typically metastasize in a predictable way, via lymphatic drainage and retroperitoneal lymph nodes (RPLN) are the most common sites for metastatic disease [2,3,4]. The lungs are the most frequent sites of hematogenous spread of non-seminomatous GCC (NSGCC).

Proper staging and follow-up of testicular cancer patients is essential for planning the treatment and avoiding unnecessary interventions. Approximately 70–80% of seminomas and one third of NSGCC have clinical stage I disease at diagnosis (there is no evidence of spread to either lymph nodes or other organs) [5,6]. Since clinical stage Ι TGCCs without risk factors have a low risk of relapse, most patients are offered surveillance by clinical examination, serologic studies, and cross-sectional imaging techniques every 3–6 months for the first year and then twice yearly [7]. Because of the potential harmful effects of irradiation in these young men, the follow-up must be planned carefully by keeping radiation doses “as low as reasonably achievable” and some new protocols include low-dose computed tomography (CT) examinations [8].

Magnetic resonance imaging (MRI) is known as a non-irradiating alternative to CT, providing high quality images of the whole body with excellent anatomic detail and functional information by adding diffusion weighted imaging (DWI) sequences [9]. Whole body MRI protocols (WB-MRI) have developed and used successfully in numerous oncological applications [10,11]. Yet, the role of WB-MRI in the staging and follow up of TGCC is not well studied.

We developed a fast (30-min) WB-MRI protocol without contrast material for the detection of lymph node (LN) metastasis in patients with TGCC. This study aimed to prospectively compare the diagnostic value of thoraco-abdominopelvic CT and WB-MRI in the LN staging of TGCC patients.

## 2. Materials and Methods

### 2.1. Study Population

This prospective single-center study was approved by the institutional ethical board (2017/30MAI/306). Informed consent was obtained from all patients. Forty-four patients entered the study between July 2017 and October 2020. One patient was excluded due to claustrophobia. Inclusion and exclusion criteria were: (i) patient ≥ 18 years old with first diagnosis of TGCC or patients with suspected or confirmed relapse, (ii) contraindications were incompatible MRI medical devices.

### 2.2. Imaging Protocols

The patients were imaged with thoraco-abdominopelvic CT and WB-MRI and then underwent a second pair of examinations depending on the type of cancer and disease stage between 2 and 12 months after treatment. CT and MRI examinations were performed within less than 30 days.

#### 2.2.1. Computed Tomography (CT)

All patients underwent a thoraco-abdominopelvic exam on an IQON^®^ Spectral CT scanner (Philips Healthcare, Best, The Netherlands) with a 120 kVp voltage and adapted mAs according to dose modulation. Portal phase images were obtained after intravenous contrast medium (100 mL, Xenetix 350^®^ Guerbet, Villepinte, France) in all patients, except for one with history of serious allergic reaction to iodinated contrast material. One of the patients received positive oral contrast material diluted in mineral water to a concentration of 5% (Telebrix Gastro^®^, Guerbet, Aulnay-sous-Bois, France). Patients were imaged in the supine position from the pulmonary apex to pelvic symphysis in one spiral acquisition. Radiation dose was evaluated using patient protocols [Mean volume computed tomography dose index (CTDIvol) and Dose length product (DLP)] available in our picture archiving and communication system (PACS, Carestream; Carestream Health, Rochester, New York, NY, USA). The effective dose in millisievert (mSv) was calculated using a “k factor” of 0.015, according to recommendations from International Commission of Radiological protection (ICRP) [12].

#### 2.2.2. Whole-Body Magnetic Resonance Imaging (WB-MRI)

All patients were imaged with a 3.0-Tesla MRI unit; 32 patients with a Philips Ingenia (Philips Healthcare, Amsterdam, The Netherlands) and 11 patients with a General Electric Signa Premier (General Electrics, Boston, MA, USA). The WB-MRI protocol consisted of an axial Turbo Spin Echo (TSE) T2 weighted sequence (T2W), a breath-hold coronal mDIXON three-dimensional (3D) gradient echo (GRE) T1-weighted (T1W) sequence, a free breathing axial DWI (b-values: 0, 50, 150, 1000 s/mm^2^) sequence and Apparent Diffusion Coefficient (ADC) maps. All sequences covered the body from the vertex to midthighs (4 stacks). The detailed MRI protocol is shown in Table 1. Sequence acquisition time was less than 20 min and scan time—including positioning of the patient and calibration—was 30 min.

### 2.3. Image Analysis

Two radiologists [reader 1 (R1) and reader 2 (R2), with 15 and 13 years of experience, respectively] blindly and independently reviewed the CTs and WB-MRIs for the presence of metastatic disease. They reviewed the CT and MR images in random order and in separate sessions at least 2 weeks apart, in order to prevent recall bias. Imaging criteria for the characterization of LNs as abnormal were as follows: (i) short-axis diameter larger than 10 mm on the anatomic sequences, (ii) loss of the normal oblong kidney bean shape and of the fatty hilum, and (iii) irregular outline [13].

The following LN regions were considered: distant lymph nodes (DLN) supra-diaphragmatic (cervical, axillary, hilar and mediastinal), scrotal and inguinal LNs and RPLN (para-caval, pre-caval, inter-aortocaval, pre-aortic, para-aortic, right and left supra-hilar, right and left internal/external/common iliac, right and left gonadal) [14].

Although this was not the primary purpose of our study, the readers also noted for suspicious lung, liver, kidney or other metastatic lesions.

The effectiveness of each WB-MRI sequence in detecting abnormal LNs (per-patient analysis) was assessed qualitatively. To this purpose, a five-point Likert scale was used for each patient for a given sequence. Likert scale is a rating scale used to collect responders’ attitudes and opinions. In this study Likert scale is used to assess image quality. Radiologists rated their overall impression concerning the effectiveness of every WB-MRI sequence for detection of abnormal LNs as follows: 0, very poor; 1, poor; 2, fair; 3, good; 4, very good; 5, excellent. Then a total score (TS) was calculated resulting from the sum of the scores for each sequence.

Quality of WB-MR images was assessed by measuring both the signal-to-noise ratio (SNR) and contrast-to-noise ratio (CNR) by placing regions of interest (ROIs) within the lesions and the reference tissues. As reference regions were chosen the retroperitoneal (infra-renal) and the gluteal subcutaneous fat.

### 2.4. Reference Standard

For 3 patients in whom surgical lymphadenectomy was performed, the histopathologic examination was used as a reference standard. For the remaining 40 patients, in whom biopsy or surgical resection of lesions was not performed, a best valuable comparator (BVC) was used as reference standard. BVC was established by a panel of experts, reviewing all baseline imaging studies along with the prospective systematic imaging, clinical and biologic follow-up of at least 1 year. The panel consisted of 4 radiologists (VP, SVN, LA, FL), 2 urologists (BT, JVD) and 1 oncologist (JPM), who determined in consensus the final diagnosis for each patient, i.e., metastatic or not.

### 2.5. Statistical Analysis

The primary endpoint was to assess the performance of each imaging protocol for the detection of metastasis on a per-patient basis. The protocols assessed were: CT, total WB-MRI protocol including all sequences (^total^WB-MRI = T1W + T2W + DWI), and two fast protocols including only one anatomic sequence and DWI (T1W + DWI and T2W + DWI). The analysis was performed for both readers independently. True Positive (TP), False Positive (FP), True Negative (TN), False Negative (FN), Sensitivity (Se), Specificity (Sp), Predictive Accuracy defined as Acc = (TP correctly classified + TN correctly classified)/(P + N), as well as the agreement measurement with the reference standard (based on Gwet’s AC1 coefficient) were reported. 95% confidence intervals (CI) on Se, Sp, Acc and AC1 were also provided [15].

An Exact test for paired data was performed to assess differences in Acc between CT and ^total^WB-MRI. As 3 lesion sites (RPLN, DLNs and other lesions) and two readers were considered, a Bonferroni correction was applied and a significance level of *p* < 0.0083 was considered for this test.

Inter-reader agreement was assessed for each imaging protocol using Gwet’s AC1 coefficient. Strength of agreement was interpreted as follows: slight (AC1 ≤ 0.2), fair (0.2 < AC1 ≤ 0.4), moderate (0.4 < AC1 ≤ 0.6), good (0.6 < AC1 ≤ 0.8) and very good agreement (AC1 > 0.8).

MR image quality was first assessed using a 5-point Likert scale. Measurements of SNR in the metastatic lesions and CNR (taking fat as reference) were then performed. A Kruskal-Wallis analysis followed by a paired Wilcoxon test was used to assess potential statistical differences in SNR/CNR between T1W, T2W and DWI sequences. A significance level of *p* < 0.0167 was considered for this test.

All data were analyzed using the Statsdirect statistical software version 3.1.20.

## 3. Results

### 3.1. Patient Characteristics

Clinical data are summarized in Table 2 and Supplementary Material (Flowchart). Of 43 patients, 24 had newly diagnosed TGCC and 19 were in follow-up. Their median age was 35.2 years (IQR 9.92). Nineteen patients (44.2%) were diagnosed with seminoma and 24 (55.8%) with non-seminoma GCC. Of the 43 patients, 22 patients had retroperitoneal lymph nodes (RPLN) metastases, 11 distant lymph node (DLN) metastases, 8 lung metastases and 1 liver metastasis. Concerning the metastatic lymph nodes, 28 RPLN were detected with median size value = 21 mm [18 mm; 28 mm] and 14 DLN with median size value = 15 mm [12 mm; 17 mm].

### 3.2. Inter-Reader Agreement

Measures of reproducibility are given in Table 3. Regarding CT, reproducibility was very good, regardless of the site of the lesion (all AC1 values ≥ 0.93). Regarding WB-MRI, reproducibility was also very good, regardless of both, the site of the lesion and the WB-MRI protocol used (all AC1 values ≥ 0.96).

### 3.3. Performance of CT and WB-MRI

Measures of performance are summarized in Table 4.

#### 3.3.1. RPLN Metastases 

**Figure 1:**
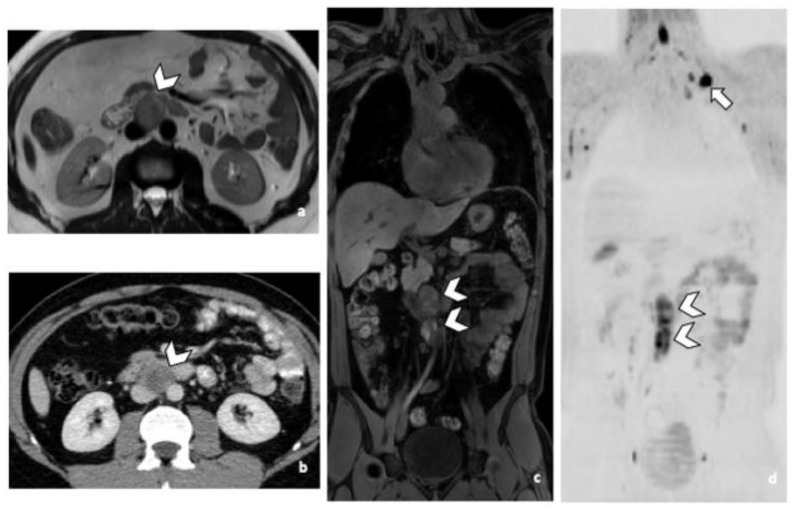
29-year-old man with NSGCC. Axial T2-weighted MR image (**a**), coronal reformatted 3D T1 GRE Fat-Sat (**c**) and high b-value DWI (inverted greyscale, b = 1000 s/mm^2^) (**d**) of MR images of the whole body and axial CT image after intravenous injection of iodinated contrast agent (Xenetix 350^®^) and per os contrast opacification (Telebrix Gastro^®^ 5%) (**b**), showing interaorticocaval enlarged pathological lymph nodes (arrowheads), identified by both readers during MRI and CT readings. The lymphadenopathies show an intermediate to high signal intensity on T2 (**a**), an heterogenous low and high signal intensity on T1 (**c**) compatible with histological component of NSGCC and a restricted Diffusion (**d**). Note the presence of a left supraclavicular lymphadenopathy visible on DWI (d: arrow). Abbreviations: NSGCC: Non-Seminomatous Germ Cell Cancer, 3D: Three Dimensional, T1 GRE: T1 Gradient Echo, DWI: Diffusion Weighted Imaging, MRI: Magnetic Resonance Imaging, CT: Computed Tomography.

**Figure 2:**
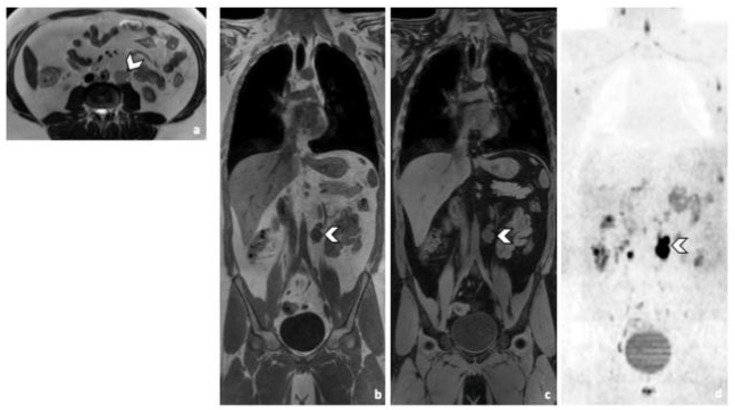
Comparison of WB sequences in a 30-year-old patient with seminoma. Axial T2-weighted (**a**) and coronal reformatted 3D T1 GRE In Phase (**b**), 3D T1 GRE Fat Sat (**c**) and high b-value DWI (inverted greyscale, b = 1000 s/mm^2^) MR images of the whole body (**d**). Detection of an enlarged pathological para-aortic lymph node (arrowhead) demonstrating an intermediate signal intensity in T2 (**a**), a hypointense homogenous signal in T1 (**b**,**c**) and a restricted Diffusion (**d**). In this case, each sequence clearly depicted the retroperitoneal lymph node. Abbreviations: 3D: Three Dimensional, T1 GRE: T1 Gradient Echo, DWI: Diffusion Weighted Imaging.

All RPLN patients were detected in both CT and WB-MRI readings by both readers (Figure 1 and Figure 2). Predictive accuracy was 100%, regardless of both, the imaging protocol and the reader. Equivalently, perfect agreement with the reference standard was observed (all AC1 values = 1.00).

#### 3.3.2. DLN Metastases

Predictive Accuracy of CT was 93%. Accuracy of WB-MRI was at least 98%, regardless of the imaging protocol. Only marginal variation in accuracy was observed between readers.

**Figure 3:**
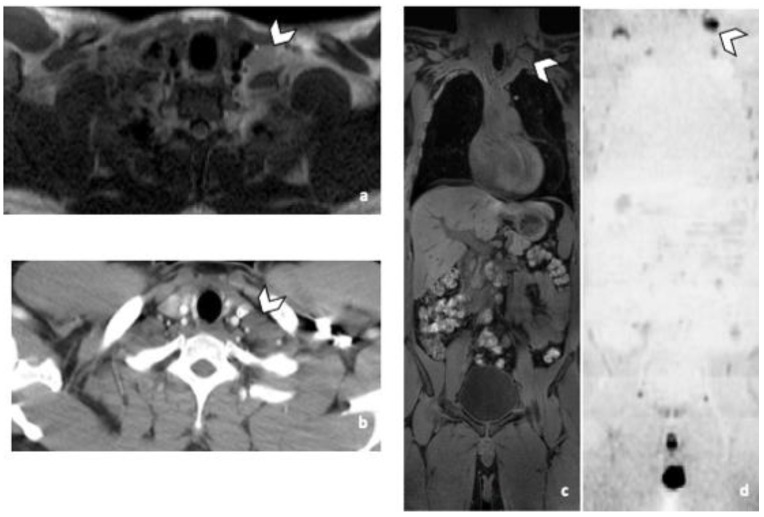
False negative supra-diaphragmatic lymph node during the CT reading in a 28-year-old man with NSGCC. Axial T2-weighted image (**a**), coronal reformatted 3D T1 GRE Fat Sat (**c**) and high b-value DWI (inverted greyscale, b = 1000 s/mm^2^) MR images of the whole body (**d**) and axial CT image after intravenous injection of iodinated contrast agent (**b**). The enlarged left supra-clavicular lymph node (arrowhead) was identified by both readers during the MRI readings; it was missed by both readers during the CT readings. Abbreviations: NSGCC: Non-Seminomatous Germ Cell Cancer, 3D: Three Dimensional, T1 GRE: T1 Gradient Echo, DWI: Diffusion Weighted Imaging, MRI: Magnetic Resonance Imaging, CT: Computed Tomography.

**Figure 4:**
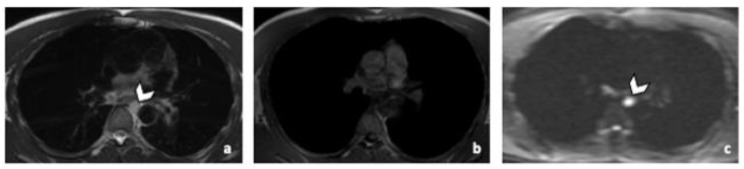
Comparison of WB sequences in a 39-year-old patient with NSGCC. Axial T2-weighted (**a**), 3D T1 GRE In Phase (**b**) and high b-value DWI (b = 1000 s/mm^2^) MR images. One of the readers missed an enlarged paraoesophageal lymph node (arrowhead) demonstrating a high signal intensity in T2 (**a**), barely visible in T1 and presenting a restricted Diffusion (**c**). Abbreviations: NSGCC: Non-Seminomatous Germ Cell Cancer, 3D: Three Dimensional, T1 GRE: T1 Gradient Echo, DWI: Diffusion Weighted Imaging.

Practically with the sole use of CT, 2 and 3 patients with supradiaphragmatic LN would have been missed depending on the reader. R1 missed 3 supra-diaphragmatic DLN and R2 two during CT readings. Both readers missed one patient, because of the non-contrast CT due to allergy contraindication. The second patient missed by both readers, had a left supraclavicular LN in the limit of the field of view of the CT (Figure 3). Its detection was difficult because of its inherent contrast. The inherent contrast is the extent to which the attenuation coefficient value of a structure differs from the one of the surrounding tissues [16]. In this case the attenuation values of the LN and the surrounding muscles are similar, the contrast is low and the lesions are not easily distinguishable. Another reason could be the arm raised position. The third DLN patient missed by one of the two readers during the CT readings, had a paraoesophageal LN, better visualized in MRI and DWI sequence (Figure 4). For R2 the specificity of both techniques was 97% for DLN because a thymic cyst was misinterpreted as a necrotic LN.

#### 3.3.3. Other Metastatic Lesions

**Figure 5:**
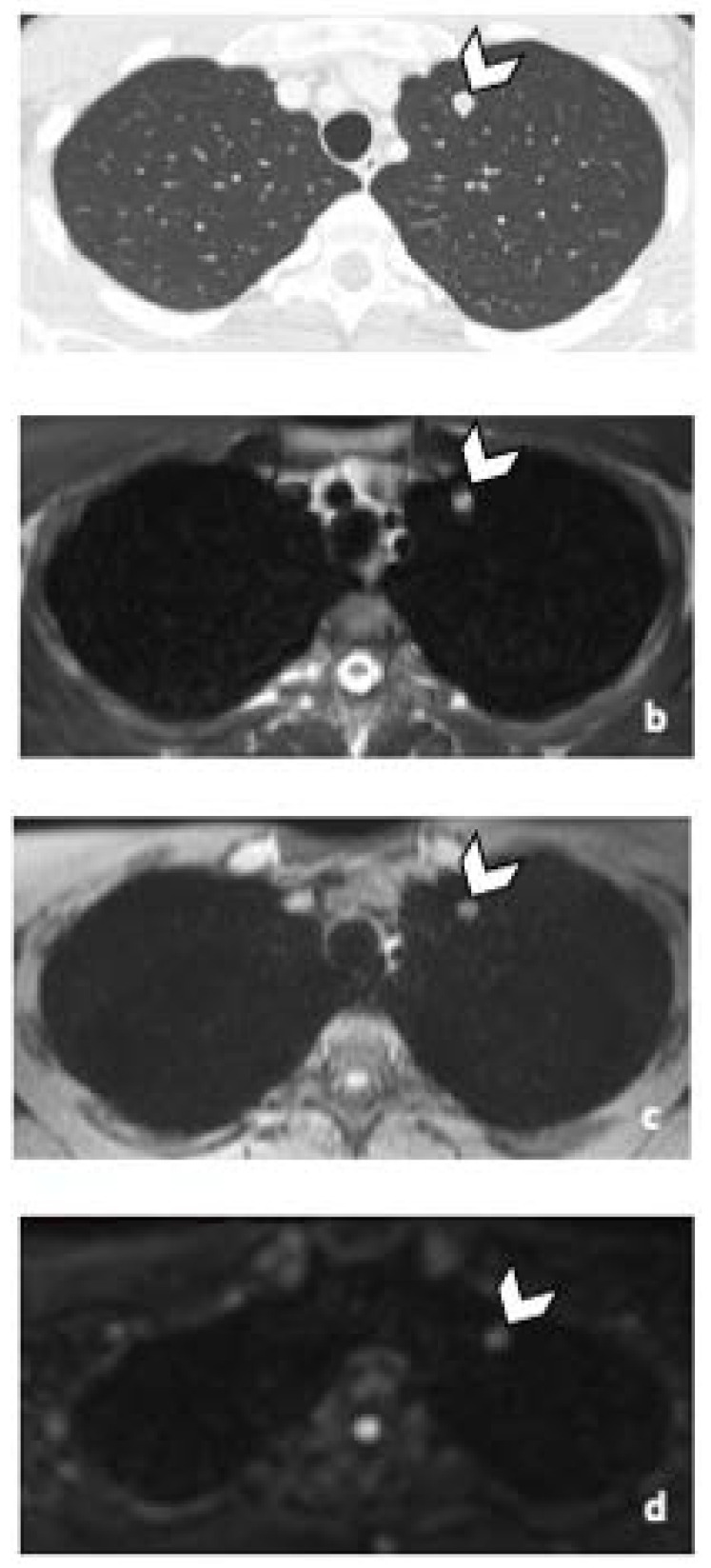
45-year-old man with seminoma. Axial CT image (**a**) and T2-weighted (**b**), 3D T1 GRE Fat-Sat (**c**) and high b-value DWI (b = 1000 s/mm^2^) (**d**) MR images. Left pulmonary nodule (arrowhead) detected by both readers during CT and MRI readings. Abbreviations: 3D: Three Dimensional, T1 GRE: T1 Gradient Echo, DWI: Diffusion Weighted Imaging, MRI: Magnetic Resonance Imaging, CT: Computed Tomography.

Predictive accuracy of CT was 98%. Accuracy of MRI was 95%, regardless of the WB-MRI protocol. No variation in accuracy was observed between readers. One patient with lung metastasis was missed during CT readings, while two were missed during WB-MRI readings by both readers, regardless of the protocol. The patients that were missed during the WB-MRI readings, had nodules that measured less than 10 mm in their largest diameter. The nodules disappeared after systematic treatment. Practically, if only WB-MRI was used, 2 patients with pulmonary metastasis would have been missed, keeping in mind that the sequences in our protocol were not developed for lung imaging (Figure 5).

During the WB-MRI readings both readers missed a patient with a pulmonary granuloma and emphysema and a patient with a Port-a-Cath thrombosis. No other significant findings were missed during the WB-MRI images.

#### 3.3.4. Comparison of Acc

Differences in Acc between CT and ^total^WB-MRI were not statistically significant, regardless of both, the site of the lesion and the reader (results from the Exact test: in RPLN, *p*^R1^ > 0.9999, *p*^R2^ > 0.9999; in DLN, *p*^R1^ = 0.2500, *p*^R2^ = 0.5000; in Other lesions, *p*^R1^ > 0.9999, *p*^R2^ > 0.9999).

### 3.4. Image Quality Assessment

#### 3.4.1. Likert Scale 

In total, there were 22 patients with RPLN and 11 DLN (Table 5). TS for each sequence could range from 0–110 for the RPLN and 0–55 for the DLN. DWI was the best sequence for the detection of RPLN followed by T2W and T1W. Likert score was at least 4 (very good) in all RPLN for DWI for both readers. Likert score was at least 3 (good) in 100% of RPLN for T2W and T1W for both readers. DWI was the best sequence followed by T2W and T1W for DLN detection. Likert score was at least 4 (very good) in all DLN for DWI for both readers and at least 3 (good) for the other two sequences.

#### 3.4.2. SNR & CNR

Quantitative measures of image quality are given in Table 6. SNR was significantly higher in DWI compared to both DIXON T1W and T2W imaging. SNR was higher in T2W imaging compared to DIXON T1W imaging (*p* < 0.0167). CNR was significantly higher in DWI compared to both, DIXON T1W and T2W imaging (*p* < 0.0167).

#### 3.4.3. Radiation Dose Analysis

DLP of the CT varied from 258.7 to 684.9 milligray*centimeter (mGy * cm) (mean 402.43 mGy * cm). Mean volume CT dose index CTDIvol was 5.44 mGy (3.5–9.5 mGy). Effective dose ranged from 3.88 to 10.3 mSv (mean 6.04 mSv).

According to the “ESMO Consensus Conference on testicular germ cell cancer: diagnosis, treatment and follow-up” and the European Association of Urology guidelines (EAU), there are three major groups of follow-up after curative treatment; firstly patients with seminoma stage I, secondly patients with NSGT stage I on active surveillance and thirdly all patients having received either adjuvant or curative treatment for good and intermediate risk metastatic disease and have achieved complete remission [7,17]. An abdominal CT or MRI are recommended for all three groups in different frequencies, when in the first group the imaging of the lung is not recommended. In the second group a simple X-ray should be used for the imaging of the chest. In the third group a thoracic CT is only recommended in case of pulmonary metastasis at diagnosis. Finally, a thoracic CT should be used in symptomatic patients, or patients with pathological RPLN or abnormal chest X-ray. The mean dose of an abdominal CT in our study was 5.11 mSv (2.7–7.52 mSv). In our institution, replacing the CT by an MRI will cut down the dose in five years by 25.503 to 30.6 mSv, depending on the patient group (Table 7).

## 4. Discussion

This prospective study assessed the performance of WB-MRI for the detection of lymph node metastases in patients with TGCC. ^total^WB-MRI including DWI + axial T2W and DIXON T1W sequences without contrast material administration, had a high predictive Acc (at least 95%, all sites of lesions considered), similar to that of CT (at least 93%, all sites considered). The strict overlap of 95% CIs associated with Acc suggests that there was no statistically significant difference in Acc between ^total^WB-MRI, T1W + DWI and T2W + DWI, regardless of the reader. All RPLN patients were detected by both thoraco-abdominopelvic CT and WB-MRI. Two and three patients with DLN were missed by R2 and R1 respectively in CT readings.

Reproducibility of the measurements was very good, regardless of the WB-MRI imaging protocol. However, as higher Likert score and SNR were observed in DWI, followed by T2W and T1W sequences, a faster MRI protocol including on T2W and DWI only may be sufficient for the LN staging of the patients with TGCCs.

TGCC primarily affects young men and carry excellent prognosis, but these patients require close follow-up by multiple cross-sectional imaging studies [17]. The benefits of frequent CTs should be weighed against the effects of radiation exposure and secondary malignancies [18]. Moreover, it is known that the use of intravenous iodinated contrast media bears a risk of adverse reactions and contrast-induced nephropathy [19]. Our WB-MRI protocol including DWI is a fast, non-irradiating technique which also avoids the need for intravenous contrast material.

The retroperitoneum is the commonest site of spread of TGCC and our study showed that WB-MRI, compared to the reference standard, missed neither RPLN nor DLN and can be used instead of thoraco-abdominopelvic CT. It has been demonstrated that MRI with DWI is as good as thoraco-abdominopelvic CT in detection of RPLN metastases [20]. An earlier study compared abdominal MRI to CT for detection of retroperitoneal metastasis in TGCC and demonstrated a sensitivity ranging from 78% to 96% [21]. Unlike our report, the aforementioned study did not use WB-MRI, focused to retroperitoneum and did not provide data for other metastatic LN sites. Moreover, the use of DWI sequences in our study may explain the better detection rates of WB-MRI in our study, as DWI is a powerful tool to detect tumoral involvement and confer added value data to anatomic WB-MRI sequences. Mosavi et al. demonstrated the feasibility of WB-MRI with DWI for the follow-up of patients with testicular cancer and the added value of DWI [22]. A recent study showed that unenhanced MRI of the abdomen and pelvis is an adequate tool for surveillance of stage I testicular cancer [23].

Chest staging is important, as TGCC may spread to supradiaphragmatic LNs and lung. For patients with NSGCC, there is a risk of pulmonary metastasis without retroperitoneal disease [24]. Our WB-MRI protocol showed excellent sensitivity and specificity in detecting supra-diaphragmatic and in general DLNs. Detection of lung nodules was not the primary focus of our study, as it is known that the conventional MR sequences still underperform in lung imaging. Our MRI protocol was not optimized for imaging of the lung parenchyma and, as expected, WB-MRI was not as sensitive as CT for the detection of lung nodules, but it was as specific. The addition of optimized sequences for the imaging of lung parenchyma, as 3D Ultrashort Echo Time or other short T2* sequences to WB-MRI, might allow better detection of lung nodules [25,26,27].

Detection of scrotal contralateral metachronous tumors is another caveat of the follow-up in TGCC [28]. Our protocol could fit the minimal requirement for detection, but not characterization of testicular lesions with the addition of a coronal T2W sequence, thus limiting the need for scrotal ultrasound [7,29].

According to the current guidelines, abdominal cross-sectional imaging is recommended for the follow up of TGCC patients after curative treatment, whereas chest imaging should be reserved only for patients at higher risk for thoracic involvement [7,17]. To put our findings into context, WB-MRI can safely replace abdominopelvic CT for the follow up of patients with TGCC after curative treatment. As we showed in the radiation dose analysis, this policy spares the patients a substantial 25 to 30.6 mSv of total radiation over a 5-year follow-up. An additional chest CT is warranted in selected patients with higher suspicion for lung metastases (lung metastases at diagnosis, symptomatic patients, patients with RPLN and patients with abnormal X-ray) [17,30]. In these cases, a low-dose CT protocol could limit the radiation exposure.

The small number of patients is a limitation of our study and larger prospective studies are needed to confirm our findings. The use of the multidisciplinary and multimodality approach as a reference-standard, in the absence of histologic proof, could be a weakness of our protocol, as it imposes a risk for differential verification biases among the patients. However, it is a standard approach in this kind of studies as the use of biopsy or surgery to obtain histopathological results would not be ethically justifiable nor feasible in clinical practice. We did not use optimized sequences for the imaging of lung parenchyma. However, conventional MR imaging is still considered suboptimal for lung imaging and was not a primary goal in our study. Finally, WB-MRI and in general MRI exams, are less available, more time consuming and costly than CT. The availability of MRI units relative to the number of inhabitants depends on the country. Moreover, to obtain WB-MRI exams, specific antennas, software, and of course specialized personnel are needed. As a result, there are countries, mainly in the developing world, in which the use of MRI is more limited due to the lack of infrastructure and the important costs, and the CT is a much more approachable imaging technique. A major future goal is to reduce the duration of the MRI acquisitions without sacrificing the quality of image.

## 5. Conclusions

WB-MRI with DWI is an accurate, fast, non-irradiating and contrast-free technique for the detection of lymph node metastases in patients with TGCC. These results provide important insights regarding the optimal and safe imaging technique in young patients with TGCC. Future studies with development of optimized lung sequences could allow WB-MRI to be a mainstay and replace thoraco-abdominopelvic CT in the initial staging and follow up of TGCC, without compromises between safety and diagnostic accuracy.

## Figures and Tables

**Table 1:** Detailed Whole-Body MRI protocol.

Target	Thorax-Abdomen-Pelvis	Thorax-Abdomen-Pelvis	Thorax-Abdomen-Pelvis
**Acquisition**	T2W	T1W DIXON	Diffusion
**Time (min:s)**	2:45 (55 s × 3 stacks)	0:47 (19 s × 3 stacks)	11:40 (228 s × 3 stacks)
**Sequence type**	TSE	MDIXON	Diffusion
**Plane**	axial	coronal	axial
**Slice × thickness/gap**	70 × 4 mm/0	153 × 3 mm/−1.5 (overlap)	50 × 6 mm/0.1
**FOV, matrix**	400 × 300, 308 × 188	300 × 450, 200 × 300	440 × 348, 100 × 73
**TR, TE, NSA, TF (TI for Diffusion)**	788, 80, 1.74	3.6, 1.32/2.3, 1,-	6329, 66, 1, - (250)
**Specific parameters**			b-values: 0, 50, 150, 1000

Abbreviations: T2W: T2 Weighted Image, T1W: T1 Weighted Image, FOV: Field Of View, TR: Repetition Time, TE: Echo Time, NSA: Number of Signal Averages, TF: Transfer Function, TI: Inversion Time.

**Table 2:** Summary of clinical data.

Patients’ Characteristics (N = 43)	
**Age (years) at diagnosis mean (SD)**	35.16 (9.92)
**Primary Histology n (%)**	
**Seminoma**	19 (44.2)
**Non-seminoma components**	24 (55.8)
Mixed Germ Cell Tumor	9 (37.5)
Embryonal Carcinoma	16 (66.7)
Yolk Sac Tumor	6 (25)
Teratoma	10 (41.7)
Trophoblastic Tumor	7 (29.2)
**Clinical Staging n (%)**	
Stage I	21 (48.8)
Stage II	5 (11.6)
Stage III	17 (39.6)
Stage IV	0
**IGCCCG n (%)**	
Good	15 (68.2)
Intermediate	6 (27.2)
Poor	1 (4.6)
**Treatment after orchidectomy n (%)**	
Chemotherapy	21 (48.8)
Radiotherapy	1 (2.4)
Active surveillance	21 (48.8)

Abbreviations: IGCCCG: International Germ Cell Cancer Collaborative Group, SD: Standard Deviation.

**Table 3:** Diagnostic characteristics of imaging protocols. Data are reported for both readers R1 (white, SV) and R2 (grey, VP). True Positive (TP), False Positive (FP), False Negative (FN), True Negative (TN) as well as Sensibility (Se), Specificity (Sp), predictive Accuracy (Acc) and agreement with the reference standard (Gwet’s Ac1) with their 95% CI are given. Differences in Acc between CT and ^total^WB-MRI were not statistically significant, regardless of both, the site of the lesion or the reader (all *p*-values > 0.0083).

RPLN								
	TP	FP	FN	TN	Se	Sp	Acc	AC1
**CT**	22	0	0	21	100 [85; 100]	100 [84; 100]	100 [92; 100]	1.00 [1.00; 1.00]
**^total^** **WB-MRI**	22	0	0	21	100 [85; 100]	100 [84; 100]	100 [92; 100] ^1^	1.00 [1.00; 1.00]
**T1W + DWI**	22	0	0	21	100 [85; 100]	100 [84; 100]	100 [92; 100]	1.00 [1.00; 1.00]
**T2W + DWI**	22	0	0	21	100 [85; 100]	100 [84; 100]	100 [92; 100]	1.00 [1.00; 1.00]
**CT**	22	0	0	21	100 [85; 100]	100 [84; 100]	100 [92; 100]	1.00 [1.00; 1.00]
**^total^** **WB-MRI**	22	0	0	21	100 [85; 100]	100 [84; 100]	100 [92; 100] ^2^	1.00 [1.00; 1.00]
**T1W + DWI**	22	0	0	21	100 [85; 100]	100 [84; 100]	100 [92; 100]	1.00 [1.00; 1.00]
**T2W + DWI**	22	0	0	21	100 [85; 100]	100 [84; 100]	100 [92; 100]	1.00 [1.00; 1.00]
**DLN**								
	**TP**	**FP**	**FN**	**TN**	**Se**	**Sp**	**Acc**	**AC1**
**CT**	8	0	3	32	73 [39; 94]	100 [89; 100]	93 [81; 99]	0.89 [0.77; 1.02]
**^total^** **WB-MRI**	11	0	0	32	100 [72; 100]	100 [89; 100]	100 [92; 100] ^3^	1.00 [1.00; 1.00]
**T1W + DWI**	11	0	0	32	100 [72; 100]	100 [89; 100]	100 [92; 100]	1.00 [1.00; 1.00]
**T2W + DWI**	11	0	0	32	100 [72; 100]	100 [89; 100]	100 [92; 100]	1.00 [1.00; 1.00]
**CT**	9	1	2	31	82 [48; 98]	97 [84; 100]	93 [81; 99]	0.89 [0.76; 1.02]
**^total^** **WB-MRI**	11	1	0	31	100 [72; 100]	97 [84; 100]	98 [88; 100] ^4^	0.96 [0.89; 1.04]
**T1W + DWI**	11	1	0	31	100 [72; 100]	97 [84; 100]	98 [88; 100]	0.96 [0.89; 1.04]
**T2W + DWI**	11	1	0	31	100 [72; 100]	97 [84; 100]	98 [88; 100]	0.96 [0.89; 1.04]
**Other**								
	**TP**	**FP**	**FN**	**TN**	**Se**	**Sp**	**Acc**	**AC1**
**CT**	8	0	1	34	89 [52; 100]	100 [90; 100]	98 [88; 100]	0.97 [0.89; 1.04]
**^total^** **WB-MRI**	7	0	2	34	78 [40; 97]	100 [90; 100]	95 [84; 99] ^5^	0.93 [0.84; 1.03]
**T1W + DWI**	7	0	2	34	78 [40; 97]	100 [90; 100]	95 [84; 99]	0.93 [0.84; 1.03]
**T2W + DWI**	7	0	2	34	78 [40; 97]	100 [90; 100]	95 [84; 99]	0.93 [0.84; 1.03]
**CT**	8	0	1	34	89 [52, 100]	100 [90; 100]	98 [88; 100]	0.97 [0.90; 1.03]
**^total^** **WB-MRI**	7	0	2	34	78 [40; 97]	100 [90; 100]	95 [84; 99] ^6^	0.93 [0.84; 1.03]
**T1W + DWI**	7	0	2	34	78 [40; 97]	100 [90; 100]	95 [84; 99]	0.93 [0.84; 1.03]
**T2W + DWI**	7	0	2	34	78 [40; 97]	100 [90; 100]	95 [84; 99]	0.93 [0.84; 1.03]

^1^ Exact 2-sided test for comparing Acc between CT and ^total^WB-MRI: *p* > 0.9999; ^2^ *p* > 0.9999; ^3^ *p* = 0.2500; ^4^ *p* = 0.5000; ^5^ *p* > 0.9999; ^6^ *p* > 0.9999. Abbreviations: RPLN: retroperitoneal lymph nodes, DLN: Distant lymph nodes, CT: Computed Tomography, WB-MRI: Whole Body Magnetic Resonance Imaging, T2W: T2 Weighted Image, T1W: T1 Weighted Image, DWI: Diffusion Weighted Imaging.

**Table 4:** Inter-reader agreement (i.e., reproducibility of the readings) based on Gwet’s AC1 coefficient and its 95%CI.

	RPLN	DLN	Other
**CT**	1.00 [1.00; 1.00]	0.93 [0.83; 1.03]	0.93 [0.84; 1.03]
**^total^** **WB-** **MRI**	1.00 [1.00; 1.00]	0.96 [0.89; 1.04]	1.00 [1.00; 1.00]
**T1W + DWI**	1.00 [1.00; 1.00]	0.96 [0.89; 1.04]	1.00 [1.00; 1.00]
**T2W + DWI**	1.00 [1.00; 1.00]	0.96 [0.89; 1.04]	1.00 [1.00; 1.00]

Abbreviations: RPLN: retroperitoneal lymph nodes, DLN: Distant lymph nodes, CT: Computed Tomography, WB-MRI: Whole Body Magnetic Resonance Imaging, T2W: T2 Weighted Image, T1W: T1 Weighted Image, DWI: Diffusion Weighted Imaging.

**Table 5:** Comparison of effectiveness of sequences for the detection of lymph node metastasis (per lesion) using a five-point Likert scale as follows: 0, very poor; 1, poor; 2, fair; 3, good; 4, very good; 5, excellent.

RPLN (Total Number = 22)							
Likert Score							
	0	1	2	3	4	5	Total Score
	Very Poor	Poor	Fair	Good	Very Good	Excellent	
**Reader 1**							
**DWI**	0	0	0	0	5	17	105
	0%	0%	0%	0%	22.7%	77.3%	
**T2W**	0	0	0	4	13	5	89
	0%	0%	0%	18.2%	59.1%	22.7%	
**T1W DIXON**	0	0	0	12	8	2	78
	0%	0%	0%	54.5%	36.4%	9.1%	
**Reader 2**							
**DWI**	0	0	0	0	7	15	103
	0%	0%	0%	0%	31.8%	68.2%	
**T2**	0	0	0	5	11	6	89
	0%	0%	0%	22.7%	50%%	22.3%%	
**T1W DIXON**	0	0	0	11	11	0	77
	0%	0%	0%	50.0%	50.0%	0.0%	
**DLN (Total Number = 11)**							
**Likert Score**							
	**0**	**1**	**2**	**3**	**4**	**5**	**Total score**
	**very poor**	**poor**	**fair**	**good**	**very good**	**excellent**	
**Reader 1**							
**DWI**	0	0	0	0	3	8	52
	0%	0%	0%	0.0%	27.3%	72.7%	
**T2**	0	0	0	4	5	2	42
	0%	0%	0%	36.4%	45.4%	18.2%	
**T1W DIXON**	0	0	0	7	4	0	37
	0%	0%	0%	63.6%	36.4%	0.0%	
**Reader 2**							
**DWI**	0	0	0	0	3	8	52
	0%	0%	0%	0.0%	27.3%	72.7%	
**T2W**	0	0	0	6	3	2	40
	0%	0%	0%	54.5%	27.3%	18.2%	
**T1W DIXON**	0	0	0	6	4	1	39
	0%	0%	0%	54.5%	36.4%	9.1%	

Abbreviations: RPLN: retroperitoneal lymph nodes, DLN: Distant lymph nodes, T2W: T2 Weighted Image, T1W: T1 Weighted Image, DWI: Diffusion Weighted Imaging.

**Table 6:** Signal-to-noise ratio (SNR) and contrast-to-noise ratio (CNR) taking fat as reference measured in 31 lesions in 26 (patients).

	SNR	CNR
**DWI**	392 [214; 607]	335 [173; 584]
**T1W**	43.5 [36.1; 53.0]	61.2 [50.0; 80.2]
**T2W**	97.1 [61.3; 112]	84.9 [56.4; 122]
Significance level after Bonferroni correction: *p* = 0.0167		
*SNRDWI* vs. *SNRT1W: p < 0.0001*		
*SNRDWI* vs. *SNRT2W: p < 0.0001*		
*SNRT2W* vs. *SNRT1W: p < 0.0002*		
*CNRDWI* vs. *CNRT1W: p < 0.0001*		
*CNRDWI* vs. *CNRT2W: p < 0.0001*		
*CNRT2W* vs. *CNRT1W: p = 0.0982*		

Abbreviations: SNR: Signal to noise ratio, CNR: Contrast to noise ratio, T2W: T2 Weighted Image, T1W: T1 Weighted Image, DWI: Diffusion Weighted Imaging.

**Table 7:** Mean accumulated Effective dose per year, depending on the patient follow up group according to the European Society for Medical Oncology (ESMO) Consensus Conference on testicular germ cell cancer: diagnosis, treatment and follow-up” and the European Association of Urology (EAU) guidelines.

Recommended Minimum Follow up for Group 1	Year 1	Year 2	Year 3	Year 4 and 5
Abdominal CT	2 times	2 times	at 36 months	1 at 60 months
Mean accumulated Effective dose per year (mSv) *	10.2	10.2	5.1	5.1
**Recommended minimum follow up for Group 2**	**Year 1**	**Year 2**	**Year 3**	**Year 4 and 5**
Chest X-ray	2 times	2 times	1 if LVI +	At 60 months if LVI+
Abdominal CT	2 times	at 24 months	at 36 months	at 60 months (optional)
Mean accumulated Effective dose per year (mSv) **	10.201	5.101	5.1005	5.1005
**Recommended minimum follow up for Group 3**	**Year 1**	**Year 2**	**Year 3**	**Year 4 and 5**
Chest X-ray	1–2	1	1	1
Abdominal CT	1–2	at 24 months	at 36 months	at 60 months (optional)
Mean accumulated Effective dose per year (mSv)	5.1005–10.201	5.1005	5.1005	5.1005

* Mean effective dose per CT (mSv) = 5.1 mSv; ** Mean effective dose per Chest X-ray (mSv) = 0.0005 mSv; Abbreviations: mSv: millisievert, LVI: Lymphovascular invasion.

## Data Availability

The data presented in this study are available on request from the corresponding author. The data are not publicly available due to ethical and privacy reasons.

## Supplementary Materials

The following supporting information can be downloaded at: https://www.mdpi.com/article/10.3390/life12020212/s1, Supplementary Materials: Patient population flow chart.
