# Supplementary figures and images for: Whole Body MRI in the Detection of Lymph Node Metastases in Patients with Testicular Germ Cell Cancer
Source: Life (Basel). 2022 Jan 29;12(2):212. doi: 10.3390/life12020212 (PMC8875751; doi:10.3390/life12020212)

Supplementary Materials: Patient population flow chart.

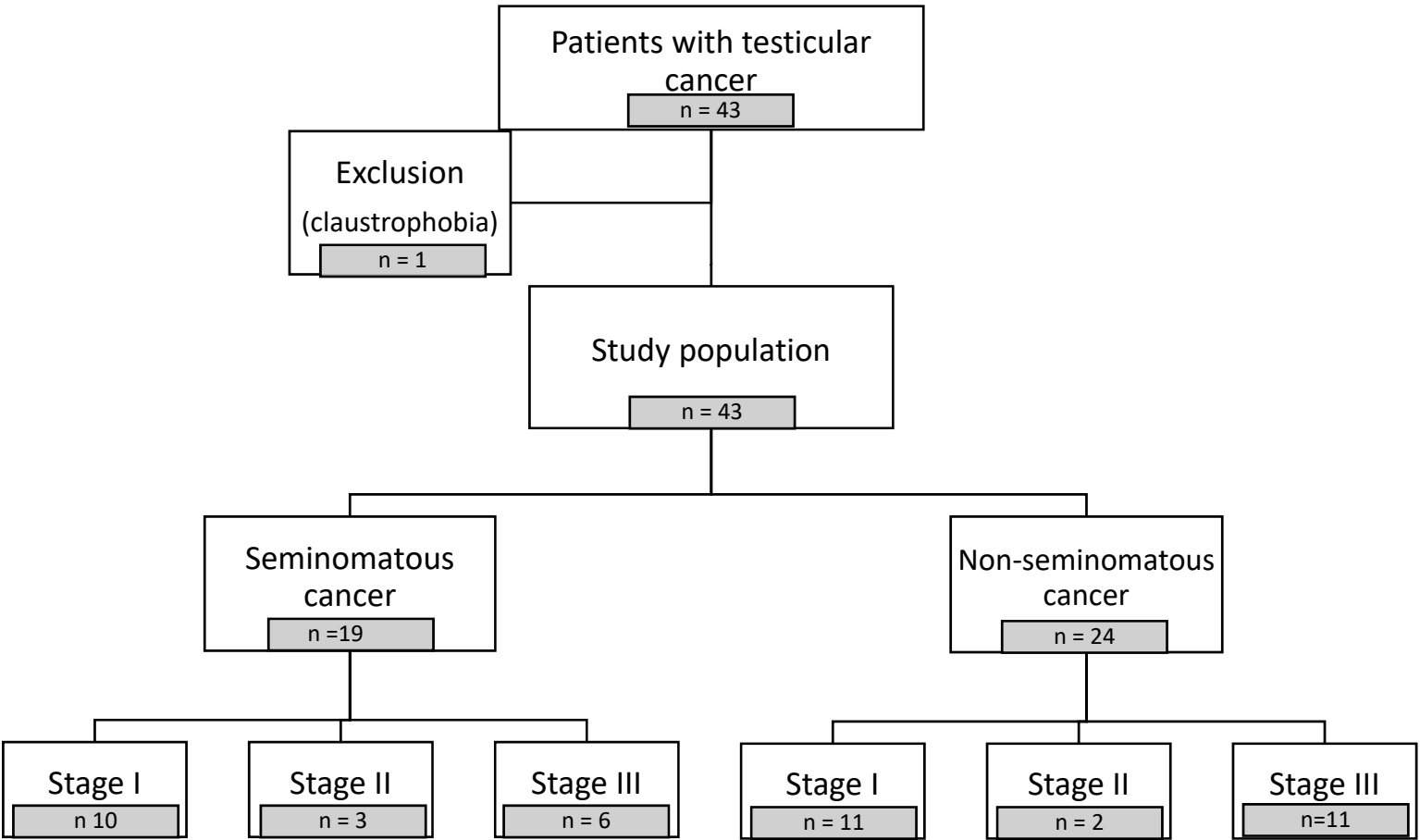

Supplement: Supplementary file 1 [file life-12-00212-s001.zip › life-1556538-supplementary.pdf]
